# The Evaluation of Simulated Environmental Degradation of Polycarbonate Filled with Inorganic and Organic Reinforcements

**DOI:** 10.3390/polym13203572

**Published:** 2021-10-16

**Authors:** Andrzej S. Swinarew, Beata Swinarew, Tomasz Flak, Hubert Okła, Marta Lenartowicz-Klik, Adrian Barylski, Magdalena Popczyk, Jadwiga Gabor, Arkadiusz Stanula

**Affiliations:** 1 Faculty of Science and Technology, University of Silesia in Katowice, 41-500 Chorzów, Poland; andrzej.swinarew@us.edu.pl (A.S.S.); tomasz.flak@us.edu.pl (T.F.); adrian.barylski@us.edu.pl (A.B.); magdalena.popczyk@us.edu.pl (M.P.); jadwiga.gabor@us.edu.pl (J.G.); 2 Institute of Sport Science, The Jerzy Kukuczka Academy of Physical Education, 40-065 Katowice, Poland; a.stanula@awf.katowice.pl; 3 Lukasiewicz Research Network—Institute for Engineering of Polymer Materials and Dyes, 87-100 Toruń, Poland; beata.swinarew@impib.lukasiewicz.gov.pl (B.S.); marta.lenartowicz-klik@impib.lukasiewicz.gov.pl (M.L.-K.)

**Keywords:** polycarbonate, aging, composite

## Abstract

This research aimed to examine the mechanical properties of polycarbonate-based composites filled with both organic and inorganic reinforcements before and after simulated environmental degradation. Series of polycarbonate-based samples were prepared in the form of thin tapes. Their rheological properties were examined. Then, the samples were exposed to artificial environmental conditions. Finally, their rheological properties were examined once more, and the results were compared with those obtained for untreated samples. This paper presents basic research on the application of inorganic fillers to polycarbonate in order to determine the influence of the filler on the behavior of the obtained material. The aim of the work was to determine the usefulness and purpose of using this type of filler in polycarbonates for applications in contact with ultraviolet radiation, especially medical applications.

## 1. Introduction

Polycarbonate is a material with excellent mechanical properties and high transparency. It is often used to produce lighting elements, both as a cover for light bulbs and, after adding appropriate inorganic additives, as an element scattering light radiation [1]. Polycarbonate is also subjected to inorganic modifications in terms of improving fire resistance [2]. There are also known studies on the influence of reinforcement in talc and glass fibers on blends of polycarbonate and acrylonitrile butadiene styrene [3]. To date, the topic of polycarbonate reinforcement has not been addressed often, and many questions remain regarding the long-term impact on the properties of the composite. It seems particularly important due to two characteristic features of polycarbonate that limit its use: low resistance to ultraviolet (UV) radiation and susceptibility to scratching [4,5].

Currently, there is an increased demand for plastics on the market, and, thus, the amount of plastic waste is growing rapidly. Polycarbonates (PCs) are a group of thermoplastic polymers that contain carbonate groups in their molecules. They are widely used in engineering because they are strong and durable materials, and some grades are optically transparent. PC is a material that can withstand multiple processing and is easy to process, form, and thermoform. The material obtained in this way is used, among other purposes, to produce milk bottles, which are more durable, lighter, and easier to clean than glass bottles. In the automotive industry, PC regranulate mixed with polyethylene terephthalate (PET) is used in the production of car bumpers. PC has high impact resistance but low scratch resistance. Therefore, the exterior polycarbonate car elements are covered with an additional protective coating. The heat-treated material is usually completely amorphous, making it highly transparent to visible light, with better light transmission than many types of glass. PC has a glass transition temperature of approx. 147 °C. PC grades with a low molecular weight are easier to form than those with higher weight grades, but their strength is lower. The hardest grades have the highest molecular weight but are more difficult to process. A big advantage in processing is that PC can undergo great plastic deformation without cracking. Therefore, it is a good material for prototype applications where transparent or electrically non-conductive parts are needed.

PC can become brittle when exposed to ionizing radiation above 25 kGy. The aging of polycarbonate is widely documented, especially when exposed to light. Photodegradation is described as a mainly two-way process [6,7,8,9]. On the one hand, the photo-Fries process is responsible for polymer decolorization [9]. On the other hand, the classic photo-oxidation mechanism causes deterioration of the mechanical properties of polycarbonate, which becomes brittle with the time of exposure [10]. This aspect is less documented, and the literature reports that the latter mechanism is mainly based on breaking macromolecular chains [6,7,8,9]. Only a few studies take into account that cross-linking reactions can take place during photo-oxidation experiments [11]. The degradation process is an irreversible, often multi-stage process, leading to clear changes in the chemical structure. During degradation, covalent bonds in the main chain of the polymer are broken, which results in the loss of functional properties of the material. Chemical and physical properties, such as molecular weight and chemical structure, change; brittleness increases; mechanical properties deteriorate; and transparency, gloss, discoloration, or changes in the surface structure occur [12,13].

There are known attempts in the literature to prevent the absorption of photons from radiation and oxygen diffusion by radio frequency magnetron sputtering of ZnO and Al_2_O_3_ ceramic thin coatings, both single and multilayer, on the PC surface [14]. It has been found that photocatalytic activity at the PC/ZnO interface is reduced in the presence of an Al_2_O_3_ intermediate layer, which limits the oxygen permeability. Nano-mechanical experiments showed that the coating systems increased both the nano-hardness of the surface and the modulus of elasticity and reduced the coefficient of friction. However, it was found that the influence of irradiation on the mechanical properties of the ceramic-coated PC surface was of secondary importance. By contrast, in the work of Bulanda et al. [15], the authors presented the results for several different composites used in 3D technology, and, on the basis of the obtained results, it was found that both the amount and type of filler significantly influenced the functional properties of the composites tested in the study.

The work presented in this article was carried out in a general context, aimed at understanding the influence of various additives on the significant mechanical properties of polycarbonate under given aging conditions. Polycarbonate is a material with highly desirable mechanical properties. Its practical use, however, is limited by its high price compared to that of alternative materials. Our work attempted to reduce polycarbonate costs by adding fillers and reducing polymer share in the material. In the research, we used hydrated magnesium silicate as a general-purpose filler for plastics, and we also proposed a proprietary polyol-polyurethane filler. The research aimed to verify the influence of this type of additive on the application properties of polycarbonates exposed to the influence of environmental conditions. The article focuses on the impact of UV light irradiation on the mechanical properties of PC to correlate modifications in the macromolecular architecture of polycarbonate with chemical changes caused by UV irradiation.

## 2. Materials and Methods

### 2.1. Base Materials

Makrolon^®^ 2600 polycarbonate, purchased from Covestro, Leverkusen, Germany, was used as the matrix material of the composite. The material is dedicated to injection technology and is characterized by a melt flow index (*MFI*) of 13 g/10 min at a weight of 1.2 kg, 300 °C, and moderate viscosity.

Rokopol^®^ D2002 (CAS 25322-69-4), purchased from PCC Rokita SA, Brzeg Dolny, Poland, was used as a compatibilizer. The material is polyoxypropylene diol with a linear structure, hydroxyl number 53–59 mg KOH/g, average molecular weight of approx. 2000 g/mol, and a density of about 1.04 g/mL.

A mixture of diphenylmethane 4,4′-diisocyanate (MDI) (CAS 101–68–8) and polymethylene polyphenyl diisocyanate (PMDI) (CAS 9016–87–9) was purchased from EPUROX Sp. z o.o., Tarnowskie Gory, Poland, and used as a linking agent for Rokopol^®^ D2002. It was a brown liquid with a density of between 1.2 and 1.3 g/cm^3^ at 25 °C and dynamic viscosity between 180 and 250 mPa*s at 25 °C.

Hydrated magnesium silicate (Mg_3_Si_4_O_10_(OH)_2_) was purchased from Zakłady Chemiczne ANSER sp. z o.o., Warsaw, Poland. The material has a suspension pH below 8.5 and a density of 2.75 g/cm^3^. The manufacturer does not specify the diameter of the fraction.

### 2.2. Composite Preparation Method

We placed 1000 g of Makrolon^®^ 2600 granules in a HSM10 planetary mixer (HORBAT, Troy, OH, USA) and mixing was started. Then, the desired amount of compatibilizing polypropylene diol was introduced and homogenized for 15 min to cover the granulate’s entire surface with liquid Rokopol^®^. In the next step, ground hydrated magnesium silicate, or an MDI/PMDI mixture, was introduced into the still stirred system in an amount corresponding to the given sample, which is described in detail in Table 1. Homogenization was continued for 20 min. The obtained mixtures were dried at the temperature of 120 °C for 6 h in polyethylene containers. Each mixture was then separately fed into the hopper of a five-zone single screw extruder L/D = 25 PLV 151 (Brabender, Duisburg, Germany,) and formed into a film using a slot die. In order to plasticize the material, the following temperatures were used in individual zones: I*—32 °C, II—240 °C, III—255 °C, IV—265 °C, V**—265 °C (*—hopper; **—extrusion head). The temperatures were based on experience in the field of industrial plastics processing. The initial temperature range was determined by data specific for pure PC [16] and was refined during extrusion based on an empirical evaluation of the parameters of the process, taking into account the extruder operating pressure and the extrudate acquisition rate.

Composite tapes with a thickness of 0.5–1.5 mm were obtained. The samples were divided into three groups to assess the effect of the MDI/PMDI mixture addition on polycarbonate, the effect of polyol content in the presence of hydrated magnesium silicate, and the effect of hydrated magnesium silicate content with 5 and 10 g of polyol. The compositions of the samples selected for further tests are presented in Table 1.

### 2.3. Aging Procedure

Under the influence of heat, water, solar radiation, stress, and many others, irreversible structural changes occur in polymers, which reduce the molecular weight or change the chemical composition and, above all, their appearance, and this often translates into a decrease in their original properties. Structural changes may result from chemical or physical changes taking place during the processing, storage and use of plastics. In order to examine the reaction of the material to the mentioned conditions, the samples were subjected to accelerated aging.

For analysis, two 2.5 cm square samples were cut from each obtained composite. One of the samples was a control sample not exposed to the simulated degradation process. The second sample was a test sample, part of the surface of which was covered so that a 1.5 × 1.5 cm fragment was exposed, including two corners of the sample and part of the center. The samples prepared in this way were subjected to the aging process, and then the color change between the appropriate test and control samples was determined.

Exposure conditions: PN-EN ISO 4892-2: 2013-06 cycle 1 modified: energy (*E*) = 74.6 W/m^2^, black standard temperature (*BST*) = 65 +/− 3 °C, temperature (*T*) chamber = 68 +/− 3 °C, relative humidity (*RH*) = 50 +/− 10%; 102 min control 300–400 nm light/18 min light + rainfall. + A dose of 238 MJ/m^2^ at 300–400 nm; time 37 days. A test of resistance to UV light with a xenon lamp was carried out in the Weather-Ometer Ci 3000+ series apparatus (Atlas Material Testing Solutions, Mt. Prospect, IL, USA). After exposure, a preliminary assessment of the changes that occurred was carried out.

### 2.4. Analytical Methods

The first test was the determination of the yellowness index of materials. The X-Rite SP 62 spectrophotometer with 8/d geometry was used to determine the yellowness index.

During the analysis, the L*, a*, and b* coordinates were obtained, marked from the CIELab color space in accordance with ISO 7724–2: 2003 Paints and varnishes. Colorimetry. Part 2: Color measurement.

Perfect white color has 100% reflectance over the entire visible spectrum and has colorimetric values of L* = 100.00, a* = 0.00, and b* = 0.00.

In the case of an element that is not perfectly white, the L* value is lower, its chromatic dimension is shifted from green to magenta (a*), and its color is shifted from blue to yellow (b*). The whiteness index determines the perfect whiteness and depends on the color shift in the area from blue to yellow (b*).

The yellowness index (YI) is a number calculated on the basis of spectrophotometric data that describe the change in the color of the test sample from transparent or white to yellow. This test is most often used to assess color changes in a material due to real or simulated outdoor exposure. YI is used to denote this quantitative change by specifying a single value.

The test technique presents a set of numbers that correlate with visual evaluations of the yellowness or whiteness of white and almost white or colorless samples viewed in daylight by an observer with normal color vision. White fabrics, paints, and plastics are some of the materials that can be described using the yellowness or whiteness indices calculated by this method.

The tribological tests were performed using the ball-disc configuration on the Anton-Paar device (tribometer—TRN, Corcelles-Cormondrèche, Switzerland). The samples were prepared in the form of discs. As counter-samples, Al_2_O_3_ balls with a diameter of ϕ 6 mm were used. The tests were carried out under conditions of technically dry friction. Tribological test conditions were load 10 N, slip speed 0.1 m/s, friction path 100 m, friction path diameter 10 mm. The tests were carried out in conditions in accordance with Versailles Project on Advanced Materials and Standards (VAMAS) and the ASTM G-99 standard [17,18]. The average area of the wear trace (P) was determined with a Mitutoyo Surftest SJ-500 profilographometer (Mitutoyo, Tokyo, Japan). Volume consumption (VW) was determined according to Formula 1:

(1) $$V_{W}=\frac{V}{F_{n}\cdot s}\left[\frac{mm^{3}}{N\cdot m}\right]$$

where *Fn*—pressure force (10 N), *s*—friction path (100 m), *V*—volume of the disc abrasion trace calculated from the formula *V* = P·2πr (mm^3^), *P*—average area of the abrasion trace determined using a profilograph (mm^2^), and *r*—radius of the friction path (5 mm). The friction coefficient was defined as the quotient of the registered friction force (*Ft*) to the applied normal force (*Fn*) [14,15].

Infrared spectroscopy measurements were performed by using IR Tracer-100 (Shimadzu, Kyoto, Japan) equipped with an attenuated total reflectance (ATR) accessory.

## 3. Results

### 3.1. Yellowing Index (YI)

**Figure 1:**
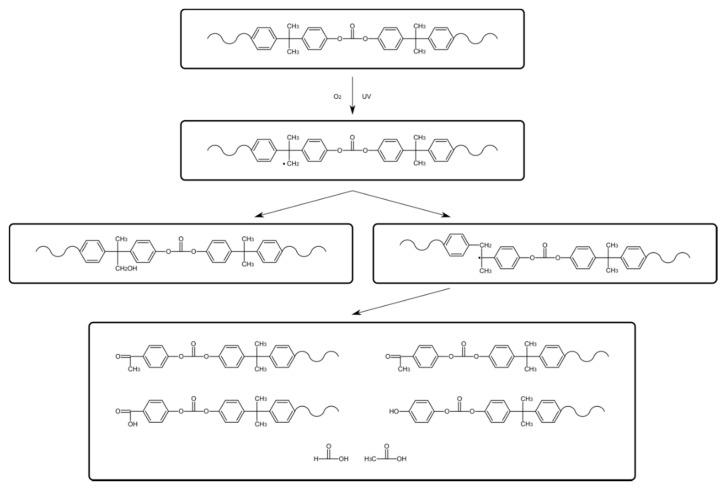
Photo-oxidative degradation of polycarbonate.

**Figure 2:**
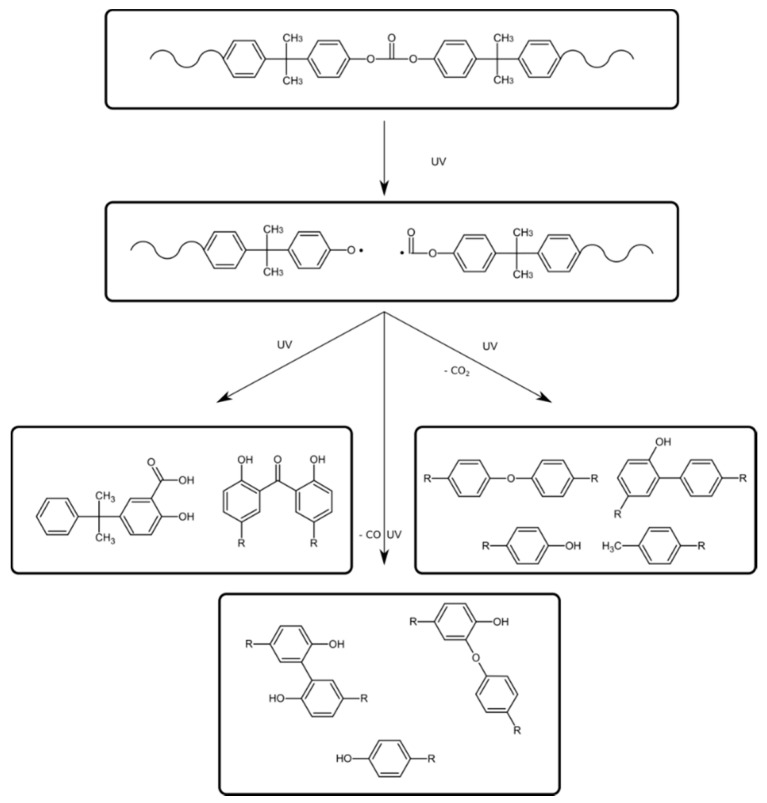
Photodegradation of polycarbonate.

Photoinduced chain breakdown leads to degradation that generates products containing salicylate groups, 2,2′-dihydroxy benzophenone and 2,2′-dihydroxy benzophenyl groups. It also leads to reorientation of the chain structure by the migration of phenyl rings. The above effects disturb the process of visible light passing through the internal structure of the chains. As a result of this disturbance, the color of the material changes (Figure 1 and Figure 2).

The first group of samples containing MDI/PMDI in their composition and without the addition of hydrated magnesium silicate is characterized by the lowest YI value. Among this group of samples, no relation between the amount of MDI/PMDI addition and the YI value was found (Table 2).

However, in the other two groups of materials, a clear increase in the YI value was observed. The addition of hydrated magnesium silicate worsened the material’s resistance to weather conditions. According to the steric structure, this is due to the effect of additional hydrogen groups in the vicinity of the Si ion. They exhibit greater chemical activity during moisturizing and under the influence of radiation.

### 3.2. FTIR Analysis

**Figure 3:**
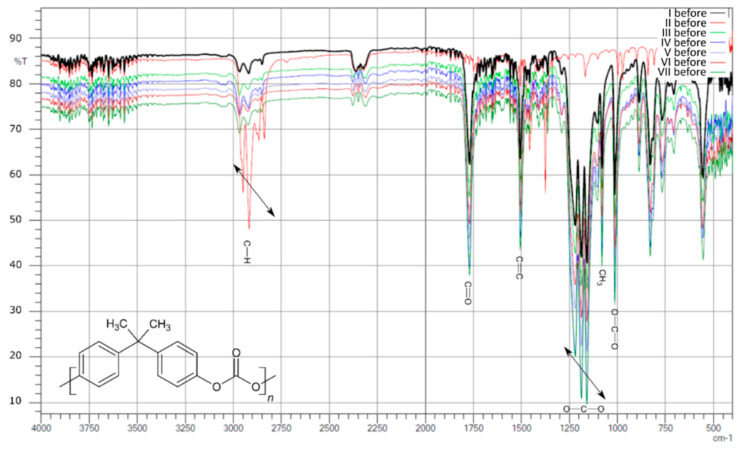
Compilation of IR spectra before aging for samples I, II, III, IV, V, VI, and VII.

Analyzing the FTIR spectra of the first group of samples (Figure 3), which did not contain hydrated magnesium silicate additives before and after aging, it can be seen, similar to YI, that the amount of addition of the MDI/PMDI mixture does not have a specific relationship with the intensity of the individual signals. This can be seen, for example, when comparing two samples with the smallest amount of additive—I and II. There is a slight difference between these two samples, and these are the two samples with the smallest addition amounts of 0.1 and 0.5, respectively, of the MDI/PMDI mixture as a polyol crosslinking agent, while the signals in sample I in the range of 1000–1300 cm^−1^ differ significantly from the other samples. This phenomenon may be related to the fact that the addition of 0.1 is imperceptible. At the same time, in other cases, its amount does not significantly affect it, and after exceeding the value of 0.5, the signals are comparable. However, there was a decrease in the intensity of some signals again as the amount of the mixture increased.

There were also observed differences in the intensity of the signals before aging in the region of 3000 cm^−1^ and the range of 1000–1750 cm^−1^. Both samples alternately show the highest or lowest intensity compared to all samples in this group. This phenomenon probably depends on factors not analyzed or taken into account, and no explanation was found in the literature.

**Figure 4:**
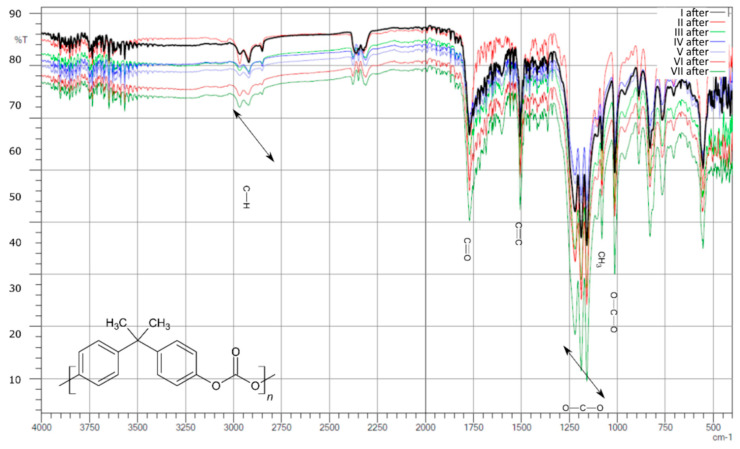
Compilation of IR spectra after aging for samples I, II, III, IV, V, VI, and VII.

After aging (Figure 4), the intensities of the individual signals in the whole group are similar, and even in sample II, processes took place whereby the intensity in the length range of about 3000 cm^−1^ decreased and was at a similar level. On the other hand, there were clear signals in the range of 1000–1300 cm^−1^ (signals from C-O-C binding), which were not observed before aging.

As a result of the comparative analysis, a clear decrease in the intensity in the region of 3000 cm^−1^, that is, signals from CH_2_ bonds, and a slight increase in the intensity of signals from carboxyl bonds in the range of 1500–1750 cm^−1^ were observed.

The second group of materials contained 3.5 g of hydrated magnesium silicate, had no MDI/PMDI additive, and differed in the amount of polyol. Samples IX and X contained 5 g of polyol, while sample VIII contained half that amount (2.5 g).

**Figure 5:**
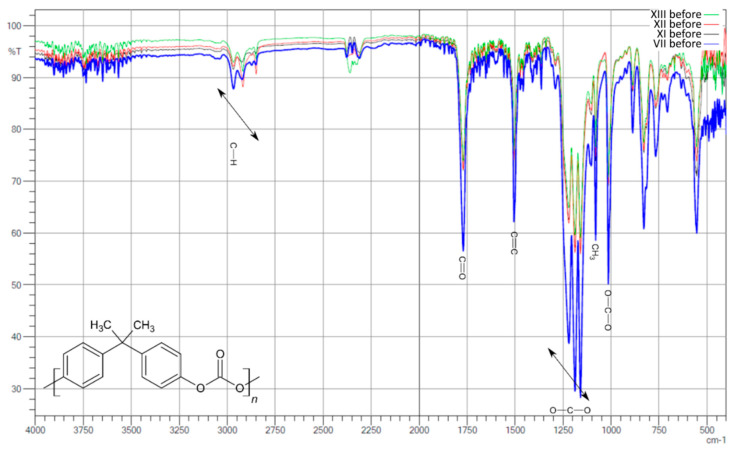
Compilation of IR spectra before aging for samples XIII, XII, XI, and VII.

In the spectrum plotted in Figure 5, it was observed that the signals for all three samples were of similar intensity. The amount of polyol only slightly affected the intensity. It was not an orderly effect, and there was no connection between the amount of polyol and the intensity of individual signals.

After aging, the question of the dependence of signals on the amount of polyol was similar. On the other hand, a clear decrease in the intensity of the signals at 1500 cm^−1^ from carboxyl groups, and 1760 cm^−1^ from C-O-C bonds, was observed. Additional signals from the carbonyl groups at 1660 cm^−1^ were observed.

**Figure 6:**
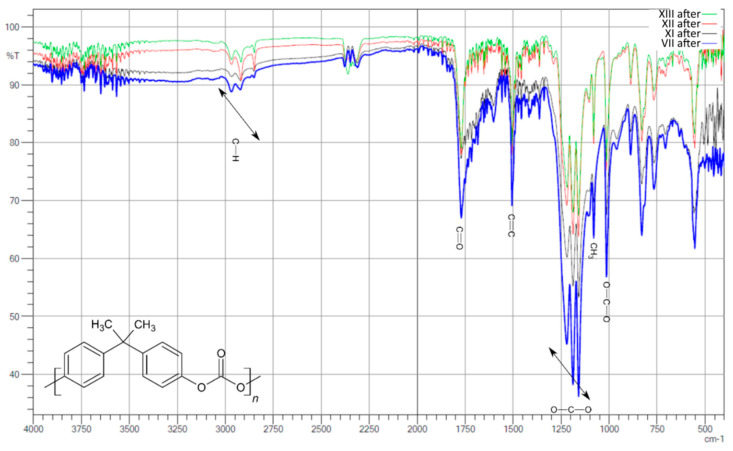
Compilation of IR spectra after aging for samples XIII, XII, XI, and VII.

The change in the absorbance at 3450, 1735, and 1838 cm^−1^ during irradiation, plotted in Figure 6, reflects the formation of hydroperoxides and alcohols, carbonyl compounds (such as ketones) and cyclic anhydrides, respectively. According to the polycarbonate chemical structure, the photo-oxidation process is expected to start by an oxidative reaction of the isopropylidene group C–CH_3_. This group is characterized by an infrared absorption at 1186 cm^−1^ [12].

### 3.3. Tribological Analysis

**Figure 7:**
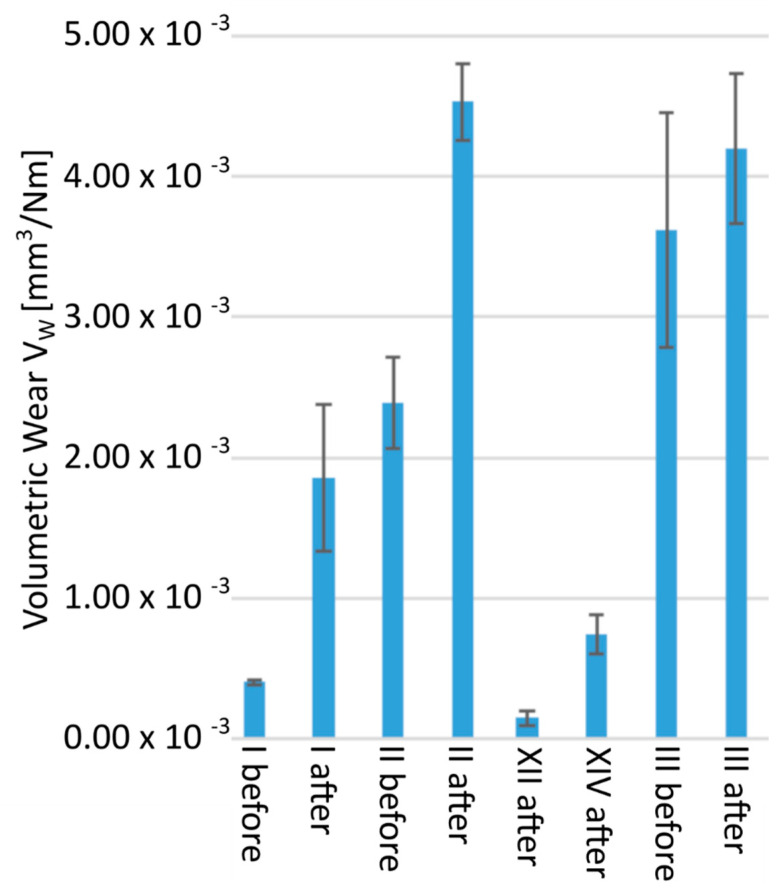
Volumetric loss Vw of the tested polymers.

**Figure 8:**
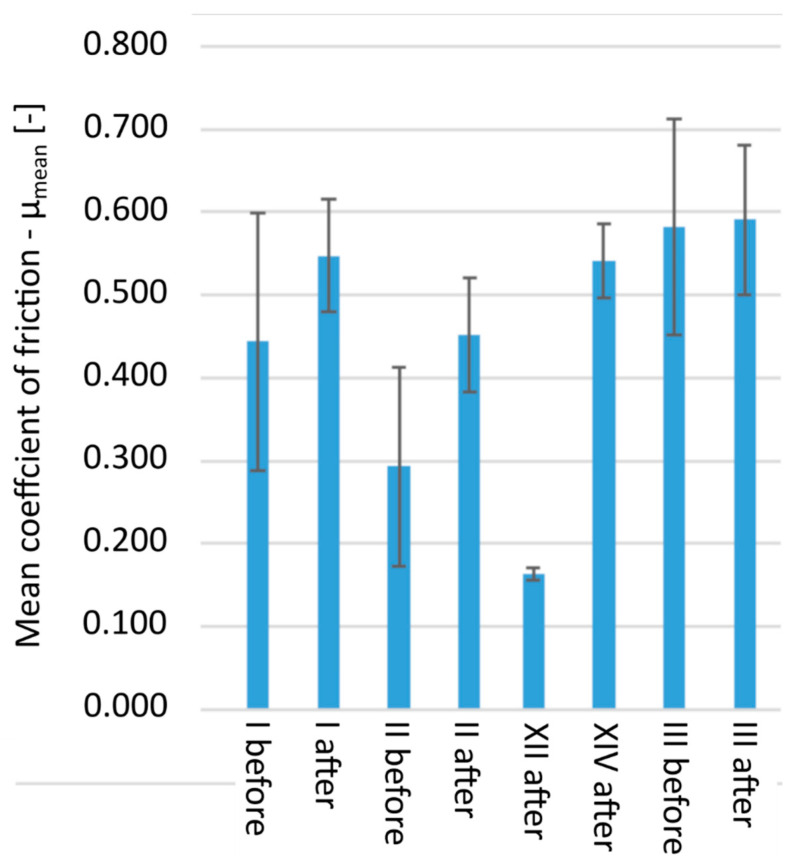
Changes in the stabilized coefficient of friction µ of the tested materials.

For the tribological tests, five pairs of samples were selected, consisting of the original sample, and subjected to aging according to the previously described procedure: I, I′; II, II′; XII, XII′; XIV, XIV′; III, III′ [Figure 7 and Figure 8], where prime symbol (′) refers to sample after aging. For samples XII and XIV, it was not possible to obtain a reliable result due to the negligible frictional wear. Samples XII′ and XIV′ were characterized by a low degree of frictional wear, which proves the good sliding properties of the hydroxylated magnesium silicate used. The increase in abrasion after aging for samples XII′ and XIV′ as compared to the materials before aging, for which the measurement could not be made, indicated a degradation of the material and a decrease in mechanical properties. The differences between samples I–I′ and II–II′ were very clearly visible. The degradation caused by exposure to ultraviolet radiation primarily led to the breakage of the polymer chains. Changing the internal structure, especially by breaking the van der Waals bonds, caused a significant increase in the consumption of polymers. For polymers I and I′, a 4.6-fold increase in volumetric wear was observed, and in the case of polymers II and II′, there was an almost 2-fold deterioration of tribological properties. The significant increase in abrasive wear was also influenced by the large increase in the stabilized coefficient of friction observed for polymers I′ and II′.

Even before aging, these materials were characterized by significantly weaker resistance to abrasion. This value increased significantly after the aging process. The wear value of sample III is very high, which proves the negative effect of the highly cross-linked polyol on the strength of the material, but a particularly interesting result is the relatively smallest difference in the values of the parameter before and after aging in the sample group III, III′. This does not match a YI of 12.5, suggesting that the polycarbonate used continued to degrade under UV influence, but the highly cross-linked polyols degraded the tribological properties to such an extent that the additional effects of aging were no longer visible.

## 4. Discussion

The demand for polycarbonate is gradually increasing in the medium term. Currently, only about 19% of polycarbonate used worldwide is processed in Europe. Unfortunately, the use of polycarbonate, despite all of its advantages, can also be a problem if it is used in items in contact with food. As previously mentioned, polycarbonate is used to produce various household items, such as CDs, packaging, glasses lenses, and smartphones. However, as shown in the study, polycarbonate does not differ from other polymers and decomposes over time, during which bisphenol A (BPA) is released. The use of polycarbonate containers for food storage is controversial, the basis of which is their hydrolysis (often referred to as leaching) at high temperatures, releasing BPA. In studies of the bioactivity of BPA derived from polycarbonates, it was proved that this compound released from polycarbonate animal cages into water at room temperature could be responsible for the enlargement of the reproductive organs of female mice [19]. However, the animal cages used in the research were made of industrial grade polycarbonate, not food grade polycarbonate. Scientists continue to try to stop this effect. Bosch et al., (2001) proved that *Geotrichum candidum* breaks down polycarbonate found in compact discs (CDs), which may have prospects in bioremediation [20]. IBM reported in 2016 that its scientists at the Almaden laboratory in San Diego had successfully solved the problem of polycarbonate product development and BPA release. Information was provided to the general public that by using a fluorine reagent, a salt of carbonic acid and heat, the scientists managed to transform the CD into a plastic with higher thermal and chemical resistance than those of the material from which it was made. During the process, the structure of the material and its strength were changed to stop the decomposition and release of BPA.

To summarize, too little is known about the degradation processes of polycarbonate and the composites that could be produced from it. The results presented in this study show that the influence of hydrated magnesium silicate on the polycarbonate degradation method is noticeable and significant. In our opinion, it is worth developing technology that facilitates the production of polycarbonate composites with mineral fillers, because, on the one hand, they limit the amount of plastic used, and, on the other hand, they facilitate the decomposition of the material, which has a positive effect on the lifetime of generated waste in the environment. The obtained polycarbonate materials, also containing partially cross-linked polyol (PO) (as a blend of PO and MDI/PMDI), did not turn yellow to the same extent as other materials tested. Hence, they extended the time that polycarbonate could be used for selected applications, such as automotive headlamps. We believe that the obtained results can become the basis for optimizing the lifetime of a product and waste-containing polycarbonate. Such works are in line with new trends in material engineering, according to which the material should be designed in such a way that it is produced, used, and disposed of without harming the natural environment. Taking into account the results of the work and the reports of the scientific world, the degradation of polycarbonate and its recycling may be safe and profitable in the future. The degradation is not always detrimental to the material; it all depends on where the material is to be used.

## Figures and Tables

**Table 1:** Compositions of the analyzed samples.

Sample ID	Polycarbonate [g]	Polyol [g]	Hydrated Magnesium Silicate [g]	MDI/PMDI Mixture
I	700	2	0	0.1
II		2	0	0.5
III		2	0	1
IV		2	0	2.5
V		2	0	1.5
VI		2	0	0.75
VII		2	0	2
VIII		2.5	3.5	0
IX		5	3.5	0
X		5	3.5	0
XI		5	7	0
XII		5	17.5	0
XIII		10	30	0
XIV		10	35	0

**Table 2:** Yellowing index of analyzed composites.

Sample ID	YI Before Aging	YI After Aging	YI Difference
I	−4.59	7.31	11.90
II	−5.56	5.28	10.84
III	4.76	17.26	12.50
IV	−6.02	4.40	10.42
V	4.85	13.37	8.52
VI	6.24	17.81	11.57
VII	1.40	11.41	10.01
IX	−10.83	22.04	32.87
X	−8.92	15.39	24.31
VIII	−5.88	29.28	35.16
XIII	1.75	29.71	27.96
XII	−4.14	32.03	36.17
XIV	2.41	38.49	36.08
XI	−10.58	19.26	29.84

## Data Availability

The data presented in this study are contained within this article.

## References

[1] Song N., Hou X., Cui S., Ba C., Jiao D., Ding P., Shi L. (2017). Polycarbonate composites: Effect of filler type and melt-blending process on the light diffusion properties. Polym. Eng. Sci..

[2] Jang K.-S. (2016). Mineral filler effect on the mechanics and flame retardancy of polycarbonate composites: Talc and kaolin. E-Polymers.

[3] Kuram E. (2019). Hybridization effect of talc/glass fiber as a filler in polycarbonate/acrylonitrile-butadiene-styrene composites. Compos. Part B Eng..

[4] West M., Ruys A., Bosi S. The Effect of the Ultraviolet Radiation Environment of LEO upon Polycarbonate Materials. Proceedings of the 43rd AIAA Aerospace Sciences Meeting and Exhibit-Meeting Papers.

[5] Liu M., Yang S., Gao C. (2020). Scratch behavior of polycarbonate by Rockwell C diamond indenter under progressive loading. Polym. Test..

[6] Factor A., Chu M.L. (1980). The role of oxygen in the photo-ageing of bisphenol-A polycarbonate. Polym. Degrad. Stab..

[7] Rivaton A., Sallet D., Lemaire J. (1983). The photochemistry of bisphenol-A polycarbonate reconsidered. Polym. Photochem..

[8] Diepens M., Gijsman P. (2007). Photodegradation of bisphenol A polycarbonate. Polym. Degrad. Stab..

[9] Pickett J.E. (2011). Influence of photo-Fries reaction products on the photodegradation of bisphenol-A polycarbonate. Polym. Degrad. Stab..

[10] Brydson J.A. (1999). Plastics Materials.

[11] Claude B., Gonon L., Duchet J., Verney V., Gardette J.L. (2004). Surface cross-linking of polycarbonate under irradiation at long wavelengths. Polym. Degrad. Stab..

[12] Collin S., Bussière P.-O., Thérias S., Lambert J.-M., Perdereau J., Gardette J.-L. (2012). Physicochemical and mechanical impacts of photo-ageing on bisphenol a polycarbonate. Polym. Degrad. Stab..

[13] Parvin M., Williams J.G. (1975). The effect of temperature on the fracture of polycarbonate. J. Mater. Sci..

[14] Mailhot B., Rivaton A., Gardette J.-L., Moustaghfir A., Tomasella E., Jacquet M., Ma X.-G., Komvopoulos K. (2006). Enhancement of the photoprotection and nanomechanical properties of polycarbonate by deposition of thin ceramic coatings. J. Appl. Phys..

[15] Bulanda K., Oleksy M., Oliwa R., Alarm clock G., Przeszłowski Ł., Fal J., Jesionowski T. (2021). Polymer Composites Based on Polycarbonate (PC) Applied to Additive Manufacturing Using Melted and Extruded Manufacturing (MEM) Technology. Polymers.

[16] https://www.plastikcity.co.uk/useful-stuff/material-melt-mould-temperatures.

[17] Czichos H., Becker S., Lexow J. (1987). Multilaboratory tribotesting: Results from the Versailles Advanced Materials and Standards programme on wear test methods. Wear.

[18] (2017). ASTM International ASTM G99-17, Standard Test Method for Wear Testing with a Pin-on-Disk Apparatus. Annu. B ASTM Stand..

[19] Bair H.E., Falcone D.R., Hellman M.Y., Johnson G.E., Kelleher P.G. (1981). Hydrolysis of polycarbonate to yield BPA. J. Appl. Polym. Sci..

[20] Bosch X. (2001). Fungus Eats CD. Nature.

